# Beirut Air Pollution and Health Effects - BAPHE study protocol and objectives

**DOI:** 10.1186/s40248-015-0016-1

**Published:** 2015-06-23

**Authors:** Myriam Mrad Nakhlé, Wehbeh Farah, Nelly Ziade, Maher Abboud, Marie-Louise Coussa-Koniski, Isabella Annesi-Maesano

**Affiliations:** Biology Department, Saint Joseph University of Beirut, Beirut, Lebanon; Physics Department, Saint Joseph University of Beirut, Beirut, Lebanon; Rheumatology Department, Hôtel Dieu de France Hospital, Saint Joseph University of Beirut, Beirut, Lebanon; Chemistry Department, Saint Joseph University of Beirut, Beirut, Lebanon; Pulmonary and Critical Care, University medical center, Rizk hospital, Beirut, Lebanon; Arcenciel, Environment Program, B.P. 165216 Beirut, Lebanon; INSERM, UMR_S 1136, Institut Pierre Louis d’Epidémiologie et de Santé Publique, Equipe EPAR, F-75013 Paris, France; Sorbonne Universités, UPMC Univ Paris 06, UMR_S 1136, Institut Pierre Louis d’Epidémiologie et de Santé Publique, Equipe EPAR, F-75013 Paris, France

**Keywords:** Air quality, Environmental health, Public health, Suspended particulate matter, Time series

## Abstract

**Background:**

Recent studies investigating the health effects of air pollution have proven an existing impact around and below international air quality guidelines and standards. These studies were based on accessible data from official registers managed by public authorities. The protocol followed in BAPHE project is described; its benefits and disadvantages are presented and discussed in this paper.

**Methods:**

Based on the review of several international studies we developed a custom made approach in BAPHE (Beirut Air Pollution and Health Effects) project in order to analyze the short term health effects of air pollution taking into consideration the lack of data availability from official sources.

**Results:**

PM_2.5_ and PM_10_ concentrations were measured in Beirut for the period starting from the 1^st^ of January 2012 to the 31^st^ of December 2012. The annual average concentrations of PM_10_ and PM_2.5_ exceeded WHO’s annual average limits by 150 % and 200 %, respectively. Health data for 11,567 individuals were collected over 12 months. A variation of hospital admission causes was observed by age categories and gender.

**Conclusions:**

This article presents a simple protocol and the descriptive results of its application in the frame of an eco-epidemiological study in Lebanon. We believe that this work is not only important on a local scale, but it could be helpful for environmental epidemiological studies in other countries.

## Background

In some countries, epidemiological studies have been very well developed and investigators highlighted the effects of moderate and low levels of air pollution on health; trying to generate evidence of interactions between pollutants and local or regional conditions [1–8]. However, in other countries, where the economic, social and political issues are the main concerns, the researches remain basic.

Several studies have demonstrated associations between air pollution and health effects. In 1952, the increase in air pollution levels has been associated with an increase in mortality and hospital admissions for respiratory diseases in London [1–3]. The analysis of individuals’ lung tissues (who died during the smog episode of 1952) revealed soot and other particles in their lungs. A recent meta-analysis of 60 studies in 35 cities all over the world shows that the increase in all-causes mortality is between 0.5 and 1.6 % for every increase of 10 μg/m^3^ of daily PM_10_ levels and 5 μg/m^3^ for PM_2.5_ levels [8]. Similarly and within the framework of APHEA (Air Pollution and Health: A European Approach multicenter study) [9] it has been estimated that an increase of 50 μg/m^3^ of PM_10_ and SO_2_ is associated respectively with an increase of 3 % and 2 % in all-causes mortality in Western Europe while these figures decline for Central Eastern Europe, to reach 0.8 % for SO_2_ and 0.6 % for the Black Smoke (BS). Other studies have more focused on vulnerable groups such as the elderly, children and newborns [5, 10, 11]. For example, in France ISAAC (International Study of Asthma and Allergies in Childhood) showed that the frequency of allergic respiratory symptoms, the sensitivity and the bronchial reactivity tests are positively associated with a high level of O_3_, SO_2_ and PM_10_ [10]. A Swiss Study on Childhood Allergy and Respiratory Symptoms with Respect to Air Pollution, Climate and Pollen (SCARPOL) conducted among children in 10 Swiss cities, has positively associated air pollution (NO_2_, SO_2_, and PM_10_) with numbers of symptoms of chronic cough such as nocturnal dry cough and bronchitis; the strongest relationship was observed for PM10 (adjusted odds ratios for chronic cough, nocturnal dry cough, and bronchitis between the most and the least polluted community for PM10 were 3.07 [95 % CI: 1.62 to 5.81], 2.88 [95 % CI: 1.69 to 4.89], and 2.17 [95 % CI: 1.21 to 4.89], respectively) [11].

Thus, hundreds of studies and methods have been developed for specific pollutants and countries. Each study has its own research protocol and health indicators respecting the considered context.

In Lebanon, the levels of pollutants in Beirut and suburbs reached the “smog” levels [12–18] visible by naked eye from the overlooking hills. Beirut has no industries with significant impact, but the development of the real estate and the increase in the car fleet, deteriorate the air quality and worsen the pollution and its effects on health.

Occasional incomplete studies were performed in Beirut trying to generate evidence of a relation between air pollution and health effects [12, 13]. However, no general protocol has been yet established to study the effects of air pollution on health. In view of lack in health system data surveillance and specific environmental conditions, which is the case of several developing countries, we decided to determine the adequate protocol estimating health effects of air pollution.

Therefore, BAPHE aims to: i) propose an adjusted methodology according to the context of Beirut; ii) study the short term association between the increase in daily levels of air pollutants and emergency admissions counts for pre-defined causes; iii) estimate the costs of air pollution health effects.

This paper describes the protocol of BAPHE. Based on international studies and on a pilot study conducted in 2 Lebanese hospitals, this study intends to define a simple methodology that can be applied while assessing health effects of air pollution in developing countries. The first phase of BAPHE started in January 2012 and ended in June 2013.

## Methods

Various methodological approaches have been used in adjusted papers. Some analyses were based on case crossover, others on cohort studies or time series methodology using specific models. Most of these studies were conducted in European and US cities where health data were computerized and well managed by public authorities (EPIDeces). Nevertheless, none of these studies suggested a protocol in a developing country.

BAPHE study is performed over a period of 3 years and it includes several phases approved by scientific and ethical committee described in the following sections.

### Selection of the study area

The study site is situated in Beirut city and identified as an urban area where the exposure of the population to atmospheric pollution could be estimated and considered as homogeneous [19].

The study is conducted in 7 hospitals which accepted to participate among 9 eligible ones. The hospitals are composed of 4 university medical centers and 3 medium to small hospitals. They are selected based on their localization in Beirut city and the existence of emergency department.

### Air pollution and potential confounder’s measurements

Pollutants are measured separately on daily basis. Then, daily mean values and daily maximum values are calculated in μg/m^3^ for each pollutant and presented by station. Periods of malfunction or calibration are also collected. The hourly average data are subsequently analyzed and validated to obtain daily average of each pollutant (average of hourly concentration). As a result, in case of failure the rule of 75 % [20] is applied to calculate daily values, otherwise the value will be missing. Moreover, the missing values are replaced by the monthly average of the period. Quality assurance procedures and instructions are implemented in each step of measurement’s process: the air pollution surveillance network of Beirut has its own internal procedure for data collection and validation and record keeping, whereas the team working on BAPHE has elaborated 3 procedures to describe, control and validate the health data collected. Firstly, the data were collected manually by very well trained medical staff, then they were entered and checked in a database by the principal investigator and finally validated by 2 senior epidemiological experts. Records are kept in hard and soft copies in 2 places.

Air pollution monitoring network provides continuous information on PM_10_, PM_2.5_ and NO_2_ whose origins are mainly from traffic, construction activities and dust storm. Relative humidity, temperature, direction and wind speed are also measured by the monitoring network of Beirut city, but, due to lack of data, the daily pollen level was not collected as for the case of several international studies [21–24].

### Health data collection

In international studies, epidemiological health data are based on their availability and are usually compiled from official registers. In France for example, data on mortality are provided by CépiDc (Centre d’épidémiologie sur les causes médicales de décès), and are classified following the International Classification of Disease (ICD 10). In all developed countries, health information system exists and the number of hospital admissions can be extracted anonymously.

Nevertheless, in some developing countries official registers managed by public authorities do not exist; consequently, investigators are obliged to get an alternative source of data by collecting information from emergency department registers, insurance database or pharmacies, …*etc.*

In BAPHE, a pilot study of 15 days was conducted in 2 out of the 7 hospitals. This pilot study helped us to adjust our protocol and to define BAPHE source and quality of health data [25].

Based on the results of the pilot study, it was decided to extract data from emergency hospitals and break them down to produce total daily hospital admissions in diagnosis categories. The total number of emergency admissions for all causes is also considered as a control in order to regulate the absence of associations with air pollution.

### Statistical analysis

The BAPHE study aims to test the association between variations of a chronological time series of health events, counted daily from a large population, and the daily variation of air pollution indicators (daily mean concentrations of pollutants). In this case, health indicators or the daily count of health events are low compared to the population exposed to the risk, thus the dependent variable follows quasi-Poisson distribution, taking into account the over dispersion of the health outcome variables. Regression model is used to study the association between the dependent variable (daily health events) and several independent variables including potential confounders (pollutant concentrations, temperature, relative humidity, day of the week, flu seasons). Based on the review of several published articles [26–31], Poisson regression is the best method to be applied in studies like BAPHE because health indicators considered are small numbers of daily event comparing with large effective population. A GAM (Generalised Additive Model) under distributed lag model framework is used. Sensitivity analysis is conducted in order to corroborate our results. The fit of the model is verified using Akaike information criterion (AIC) methods, whereas transformations on the covariates can be performed using different types of spline functions, in a way to minimize the partial autocorrelations of the corresponding residuals. Based on an autoregressive model, the calculation of the relative risk can be possible.

## Results

### Pilot study results

The results of the pilot study showed that the most important source of data in the Lebanese hospitals is the emergency department (ED) register. This study determined the appropriate health indicators for BAPHE and created a classification methodology to collect health data [25].

### General study results- phase I

#### Air pollution and meteorological measurements

PM_2.5_ and PM_10_ concentrations were measured in Beirut for the period starting from the 1^st^ of January 2012 to the 31^st^ of December 2012. Table 1 shows that the daily average concentration of PM_10_ and PM_2.5_ has been found to be of 51.3 ± 33.1 μg.m^−3^ and 30.3 ± 19.4 μg.m^−3^ respectively, with corresponding maximum values of 359.7 and 208.6 μg.m^−3^. The mean concentration of coarse particles (PM_10-2.5_) was found to be 41 % of the average PM_10_, suggesting that the site is also influenced by re-suspended surface dust and soil. The mean PM_2.5_/PM_10_ ratio for the entire study period was 0.61 ± 0.12. This average indicates that in Beirut about 61 % of PM_10_ is made up of PM_2.5_, *i.e.* fine particles comprise a large fraction in PM_10_ and indicate the larger health impacts that can be caused by such particles. A total of 133 daily PM_10_ and 129 daily PM_2.5_ concentrations were higher than the limit value of the WHO, which represents 39 % and 38 % of the sampling days, respectively. The highest daily averages of the PM_10_ and PM_2.5_ were observed in spring and summer (March to August).

**Table 1:** Statistics for daily PM_10_, PM_2.5_ and PM_10-2.5_ concentrations during January 2012 – December 2012

Pollutant	N total (days)	Min (μg/m^3^)	Max (μg/m^3^)	Mean (μg/m^3^)
PM_10_	343	16.0	359.7	51.3 ± 33.1
PM_2.5_	343	4.5	208.6	30.3 ± 19.4
PM_10-2.5_	343	1.0	157.7	21.0 ± 17.3

Daily mean temperatures and relative humidity were calculated by season. Table 2 shows that the highest levels of temperature are in summer and the wettest weather is in spring where, as mentioned above, dust events occur. Knowing that Table 2 shows the average of meteorological data, it is important to know that temperature could reach 30-35 °C in summer season.

**Table 2:** Temperature (°C) & relative humidity (%) daily measurements statistics by season

Season	N total	Temperature (°C)			Relative humidity (%)		
		Min	Max	Mean	Min	Max	Mean
Winter	105.00	7.21	17.83	12.47	36.50	71.54	47.47
Spring	92.00	10.75	25.46	19.64	33.50	75.04	57.35
Summer	92.00	24.54	28.61	26.75	43.33	73.83	52.72
Autumn	52.00	19.00	28.50	23.65	32.58	73.96	52.22

### Health data

**Fig. 1:**
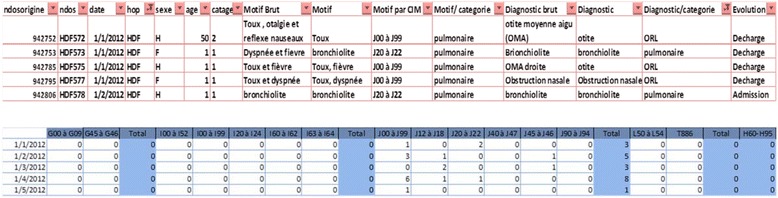
Example of database producing total daily count of emergency hospital admissions

Information on 11,567 individuals were collected over 12 months for the first phase of the study. Table 3 presents a summary description of health indicators collected from 7 hospitals in Beirut to be used in the BAPHE project. These data were subject to quality analysis and adequacy before being used in BAPHE [25]. Specific causes of admission, mainly diseases of the respiratory, cardiovascular and cerebrovascular systems in addition to skin allergic diseases have been chosen as most relevant outcomes of air pollution based on literature review [9, 19, 32]. Since we are dealing with personal information, data included only: patient number, date of admission, first diagnosis, final diagnosis, treatment and patient progress (hospitalized, transferred or discharged). International code diseases are added for the patient first complaint and the final diagnosis (Fig. 1). Data from 7 hospitals had been broken down to produce total daily hospital admissions in diagnosis categories as per Fig. 1. Data on influenza admissions were collected but we could not use them since the diagnosis process in the Lebanese hospitals is not computerized and several causes of illness may be confused with influenza. So we integrated this confounder in our model to control for flu season [33].

**Table 3:** Non-exhaustive list of health indicators and corresponding first diagnosis

Health indicators	First diagnosis to be collected
Cerebrovascular diseases	CVA, aphasia, hemiplegia, stroke, *etc.*
Circulatory Diseases	Chest pain, Thoracic pain, Thoracic Oppression, retrosternal burn, angina, unstable Angina, myocardial infarction, Acute Pulmonary Oedema (APO), sub APO *etc.*
Respiratory Disease	Dyspnea, Tachypnea, Polypnea, difficulty in breathing, Desaturation, COPD Exacerbation, asthma, Bronchospasm, Pneumonia, Bronchitis, Bronchiolitis, Bronchopneumonia, cough *etc.*
Skin Allergic Diseases	Urticaria, facial oedema, pruritis, rash, anaphylaxia *etc.*
Digestive system diseases	Considered as a control

We also checked some health figures since we searched for a homogenous study area. Therefore, we verified the mortality rate in 2010 which was 5.4 per 1,000 almost unchanged since 2006. Thus, we considered that our population is almost stable [34]. For tobacco addiction, the results can be considered stable since we studied the short term effects of air pollution and people will not change instantly.

**Fig. 2:**
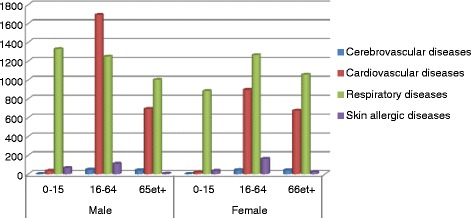
Total counts of emergency hospital admissions per gender and age groups

Figure 2 shows the variation of hospital admission causes by age categories and gender. The respiratory system diseases affect all age groups. If we consider the vulnerable categories apart, we realize that the number of male aged less than 15 years are higher than female of the same category. For elderly, little difference exists between the two genders for the same causes of admission. In adults category men are more affected by cardiovascular diseases than women who developed more respiratory diseases during the study period and according to the sample studied.

The application of an autoregressive Poisson model was used to evaluate the association between daily concentrations of particulate matter and respiratory and cardiovascular emergency hospital admissions after controlling for confounders. All variables were measured during 1 year from January 2012 to December 2012. Relative risks of admissions for respiratory and cardiovascular diseases were calculated for an increase of 10 μg.m^−3^ in pollutant concentrations. Total respiratory admissions were significantly associated with the levels of PM_10_(1.012 [95 % CI 1.004–1.02]) per 10 μg.m^−3^ rise in daily mean pollutant concentration for PM_10_ and 1.016 [95 % CI 1.000–1.032] for PM_2.5_ on the same day. A nearly significant association was found between particles and total circulatory admissions for adults and elderly groups in the same day. The results of the regression model were subject to a detailed article [35].

### Training of individuals

To extract information from emergency hospitals’ registers we trained individuals in the 7 participating hospitals to collect accurate data. Several causes of emergency admissions can be considered, an example of diagnosis category and subcategory is described in Table 3.

## Discussion

The total number of case extracted from ED registers are remarkable knowing that it is the first time in Lebanon that an environmental epidemiological study concerns such large number of individuals as BAPHE. Other obtained results are quiet impressive, especially the number of exceeding days.

If we compare our results to a population based study in Ontario Canada, the sex differences in hospital admissions from emergency departments in asthmatic adults, showed that women were more likely to be admitted than men in ED. Following [36], the higher admission rates in women may be related to sex differences in the subjective perception of dyspnea, management of asthma by ED physician, or inadequate ambulatory care strategies in women. In the same concept of disease and illness’ perception the difference between adults for cardiovascular diseases admissions can be explained may be by the same reason in Lebanon. Further analysis will clarify us more if the sex differences in hospital admissions have an impact on the association between air pollution and health effects.

However, our study presents some weaknesses. The disadvantage of studies like BAPHE involves in data collection since the health system in Lebanese hospital is not yet computerized. The hard part of the project was to collect the data manually. On another hand, this operation allows an efficient control of data through database checking by 3 different qualified experts.

If we consider the fact that time series studies have lack of individual measurements in exposure and outcomes and the possible presence of unmeasured confounders, this could be a weak point for our study. However, in BAPHE as in international studies we worked on the short term effects by using long series of small units (days) to minimize errors [26, 27]. In that design the population serves as its own control over time and confounders can only be factors varying according to the small time-units from day to day like cigarette smoking or other confounding factors.

## Conclusions

For decades, the effects of air pollution on human health were subject of many researches in public health in European, US and Asia cities. Several techniques and methods of analysis have been developed. Thus, this article presents a simple protocol and the descriptive results of its application in the frame of an eco-epidemiological study in Lebanon. We believe that this work is not only important on a local scale, but it could be helpful for environmental epidemiological studies in other countries. We have indicated how to collect reliable data on air pollution and health outcomes in order to investigate health impact of air pollution at urban level.

Therefore, BAPHE will provide a unique opportunity to assess the relation between high levels of air pollution and health effects in a country like Lebanon.

Finally, our results will contribute to set common targets and objectives for air quality and public health, guidelines to prevent, reduce or mitigate the health effects of air pollution in the city of Beirut.

## Abbreviations

BAPHE: Beirut air pollution and health effects
PM_2.5_: Particulate matter less than 2.5 μg.m^−3^
PM_10_: Particulate matter less than 10 μg.m^−3^
WHO: World Health Organization
CépiDc: Centre d’épidémiologie sur les causes médicales de décès
NAAQS: National Ambient Air Quality Standards
GAM: Generalised additive model
ED: Emergency Department
EPAR: EPidemiology of the allergic and respiratory disease
